# A Comparative Study between Use of Arthroscopic Lavage and Arthrocentesis of Temporomandibular Joint Based on Computational Fluid Dynamics Analysis

**DOI:** 10.1371/journal.pone.0078953

**Published:** 2013-11-01

**Authors:** Yue Xu, Han Lin, Ping Zhu, Wenyan Zhou, Yi Han, Youhua Zheng, Zhiguang Zhang

**Affiliations:** 1 Guanghua School of Stomatology, Sun Yat-sen University, Guangzhou, Guangdong, People’s Republic of China; 2 Department of Applied Mechanics and Engineering, Sun Yat-sen University, Guangzhou, People’s Republic of China; 3 Shanghai Ninth People's Hospital Affiliated Shanghai Jiao Tong University School of Medicine, Shanghai, People’s Republic of China; Mayo Clinic College of Medicine, United States of America

## Abstract

Arthroscopic lavage and arthrocentesis, performed with different inner-diameter lavage needles, are the current minimally invasive techniques used in temporomandibular joint disc displacement (TMJ-DD) for pain reduction and functional improvement. In the current study, we aimed to explore the biomechanical influence and explain the diverse clinical outcomes of these two approaches with computational fluid dynamics. Data was retrospectively analyzed from 78 cases that had undergone arthroscopic lavage or arthrocentesis for TMJ-DD from 2002 to 2010. Four types of finite volume models, featuring irrigation needles of different diameters, were constructed based on computed tomography images. We investigated the flow pattern and pressure distribution of lavage fluid secondary to caliber-varying needles. Our results demonstrated that the size of outflow portal was the critical factor in determining irrigated flow rate, with a larger inflow portal and a smaller outflow portal leading to higher intra-articular pressure. This was consistent with clinical data suggesting that increasing the mouth opening and maximal contra-lateral movement led to better outcomes following arthroscopic lavage. The findings of this study could be useful for choosing the lavage apparatus according to the main complaint of pain, or limited mouth opening, and examination of joint movements.

## Introduction

Over the past 15 years, arthroscopic surgery, arthrocentesis, and physical therapy have commonly been used as therapeutic interventions for permanent temporomandibular joint disc displacement (TMJ-DD) [1]. Lavage of the TMJ was first conducted using arthroscopy by Ohnishi [2]. Subsequently it was thought that visualization of the joint is not necessary to accomplish the treatment objectives; thus, arthrocentesis alone can be used as a modification of TMJ arthroscopic lavage in treatment of this condition [3,4]. The therapeutic effect of joint lavage is attributed to removal of inflammatory cells, cytokines, and degradation products of the inflamed synovium, facilitating the anti-inflammatory effects of intra-articular corticosteroid administration [5]. It appears to be a safe and effective method for reducing pain and increasing mandibular range of motion in approximately 86% of patients [6]. 

The exact technique of joint lavage reported in the literature varies considerably. The fundamental principle that is accepted in most methods, using either arthrocentesis or arthroscopic lavage, is the spatial orientation while placing the needles. It is recommended that two needles be inserted along the Canthal Tragal line. The first needle, the inflow portal, is placed into the upper joint compartment of the TMJ, and the second needle is placed anterior to the first to allow effective lavage of the joint. Due to the limited space of the upper joint compartment and technically challenging nature of the procedure, arthrocentesis using two smaller diameter needles, or even a single needle [7], is preferred over the traditional procedure of TMJ arthroscopic lavage. The major difference between arthroscopic lavage and arthrocentesis is that the surgical apparatus utilizes needles with different diameters. However, the biomechanical features of the irrigated fluid associated with the needles’ inner diameters, such as change in fluid flow and pressure distribution patterns, have not been intensively studied. A better understanding of these features could provide insights to the optimal surgical procedure for TMJ lavage, and offer a theoretical basis for improving clinical outcome.

Some studies have suggested that both arthrocentesis and arthroscopic lavage provide significant reduction in pain and increase maximal mouth opening on follow up [8-10]. Arthroscopy shows better outcomes in terms of improvement in functional outcome, whereas there is no difference in degree of pain control with either of the techniques. Therefore, because arthrocentesis is technically easier to perform compared to arthroscopic lavage, arthrocentesis is highly recommended to relieve pain in patients with painful clicking in the TMJ that does not respond to non-invasive medical management [11]. 

In this study, we first analyzed the pattern of fluid flow and pressure distribution during TMJ lavage to explain the biomechanical rationale behind the postoperative benefit in lavage with arthroscopy compared to athrocentesis. Four finite volume fluid dynamic models, with various irrigation needle modifications, were also used to simulate the lavage process and we determined the optimal needle diameter for this procedure.

## Materials and Methods

### Ethics statement

The institutional review board of the Sun Yat-sen University approved the study protocol of this retrospective study. All patients in the study group had consented to be a part of trial after clinical briefing on methodology, and signed informed consent documents were obtained. The study design was approved by the institutional ethics board of the Hospital of Stomatology, Sun Yat-sen University. 

### Patient population

The study cohort consisted of consecutive patients presenting with TMJ-DD who met 1991 research diagnostic criteria for temporomandibular joint disorders (RDC/TMD) [12]. Patients were excluded from the study if they had any of the followings: 1) masticatory muscles disease; 2) TMJ osteoarthritis; 3) pregnancy; 4) breastfeeding; 5) malignancy; 6) previous treatment with arthroscopy or arthrocentesis. 

Intraoperative and perioperative data that were included in the database included operative procedure, dosage, lavage pressure, intra-operative intra-articular pressure (IAP) and perioperative complications. Patient records and radiographs were retrospectively reviewed for pertinent data. The first group consisted of 37 patients who underwent arthroscopic lavage from 2002 to 2006. The other group consisted of 41 patients who underwent arthrocentesis from 2006 to 2010. Gender distribution (average age ± standard deviation) of the two lavage groups was: (arthroscopic group) 29 females (37 ± 16 yrs), 8 males (30 ± 12 yrs); (arthrocentesis group) 30 females (39 ± 11 yrs), 11 males (34 ± 6 yrs). Records of follow-up of all the patients over a 3-month period were obtained and analyzed. The same clinician evaluated each patient for the following criteria: joint pain using a visual analogue scale (VAS) (1–100 mm), joint noises (clicking, crepitus or none), history of locking, duration of the symptoms, maximal inter-incisal opening (MIO) and maximal contra-lateral movement (ML) [13]. Assessment of the therapeutic success of an individual procedure was based on the following criteria: an MIO no less than 3.0 mm in breadth; minimal or little postoperative pain (VAS less than 30.0 mm in breadth); few or no functional or dietary restrictions.

### Surgical procedure

All operations were performed under local anaesthesia by the same surgeon using the same type of instruments in one hospital. A double portal lavage technique [14] was used in all cases, the inflow needle was inserted into the superior joint space using the superior posterolateral approach, and the outflow portal was introduced in superior anterolateral route. For the arthroscopic procedure, two cannulas of 2.4 mm internal diameter were applied, while two 18-gauge needles of 0.8 mm internal diameter were used for the arthrocentesis procedure. In both the groups, the inflow and outflow needle used were of the same size. During the operative process, the joint was irrigated with 500 ml lactated Ringer’s solution under the inflow fluid pressure of 27.575kPa based on the recommendations of previous studies [15]. Both the lavage fluid pressure and IAP were monitored continuously during the procedure. After complete irrigation, 1 mL of sodium hyaluronate (10 mg/mL) was injected into the joint through one of the needles.

### Fluid Dynamics Analyses

All patients were enrolled in this study for use as the reconstruction models. Computed tomography (CT) arthrography was performed for each patient and sections in axial and sagittal planes of the TMJ zone (on the symptomatic side) were obtained prior to joint lavage. Based on the findings and recommendations of our previous works [16,17], the boundary was extracted from the transverse slices of the TMJ using an interactive medical image system (3D-DOCTOR; Able Software, Lexington, MA, USA) and four polygon-based models for different lavage processes of the closed-jaw position were reconstructed. The needle of smaller diameter, 0.8mm, was represented by the capital letter “N”, while the cannula of 2.4 mm inner diameter was denoted by “K”. In the following acronyms, the initial letter represents the diameter of the inflow needle and the later one represents the outflow needle. The four types of simulated models consisted of NN, KK, NK, and KN designs. NN and KK groups were used to simulate arthrocentesis and arthroscopic lavage, respectively. To further evaluate variations in TMJ lavage, apparatus modifications in the inflow and outflow portal, i.e. NK and KN types, were also simulated and considered.

**Figure 1:**
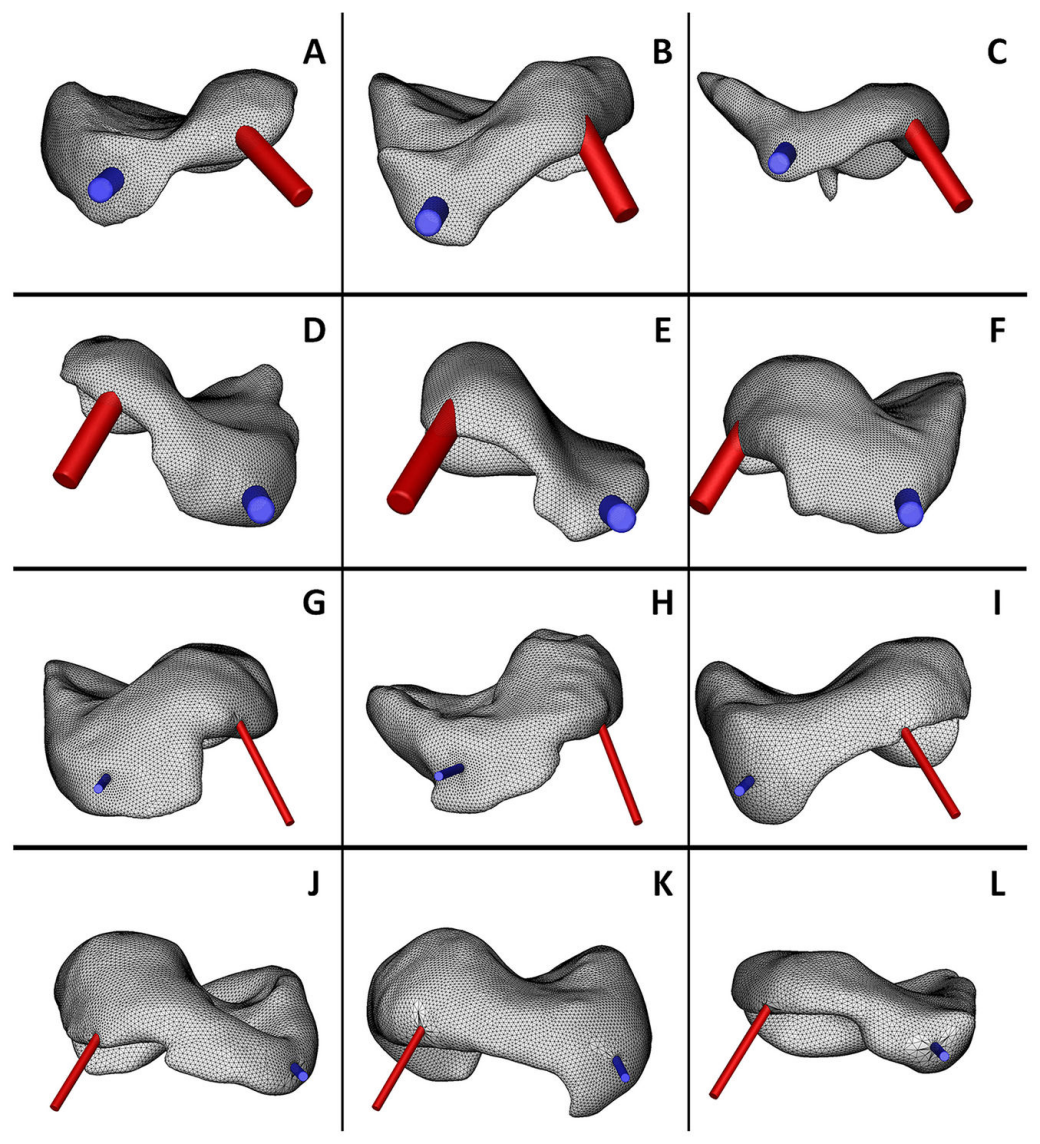
Three dimensional finite volume models of upper TMJ compartment on symptomatic side (A-C and G-I, left side; D-F and J-L, right side; 12 examples in 78 patients). The lavage process for patients was conducted with (A-F) arthroscopic procedure and (G-L) arthrocentesis procedure. The inflow and outflow portal are indicated by the red tubule and blue tubule, respectively.

The lavage fluid vessel and outer cannula wall were excluded from the computational domain for simplicity (Figure 1). Irrigated fluid was transmitted from superior posterolateral to the anterolateral needle through the superior joint space. The inflow fluid pressure was set at 27.57 kPa in the closed-jaw position [15]. We referred to our previous modeling works [16,17] for constructing the upper compartment of TMJ and simulating the lavage fluid flow in the compartment. Commercial flow modeling software (GAMBIT 2.1.30; ANSYS Inc., New York, NY, USA) was used to mesh the model and set up the boundary conditions and fluid-solid conditions. The meshed data were transferred into Fluent 6.1 software (ANSYS Inc., New York, NY, USA) to set up computational models and all parameters. The lavage fluid was assumed to be a homogeneous and incompressible Newtonian fluid with a density of 1000 kg/m^3^. A three dimensional (3D) continuous equation for an incompressible fluid and the Navier-Stokes equation [18] were used as the flow equations. 

### Statistical Analysis

Statistical analyses were performed to assess the difference between the effect of TMJ arthroscopic lavage and TMJ arthrocentesis. Results are expressed as means ± standard deviation, unless otherwise specified. Paired-Samples *t*-test was used to compare differences between groups and within groups for the clinical effect of TMJ arthroscopic lavage and TMJ arthrocentesis. The data obtained from simulated models were subjected to Repeated Measure ANOVA to test for differences between groups, and if a significant difference was indicated according to Wilks’ Lambda, the groups were further pairwise compared by Bonferroni method. *P*-values < 0.05 were considered significant. Statistical calculations were performed by using SPSS version 16.0 (SPSS, Inc., Chicago, IL). 

## Results

**Figure 2:**
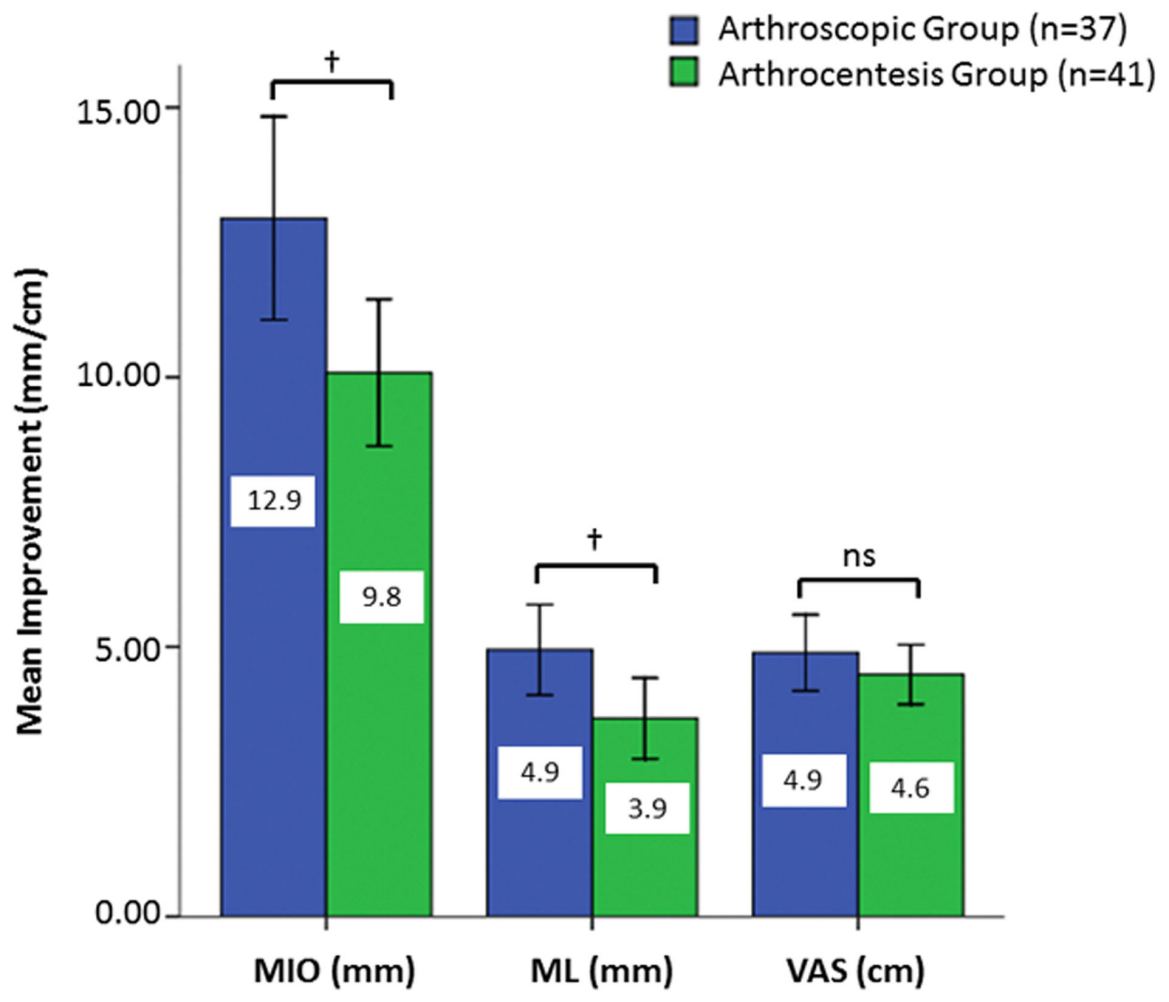
Graphs showing the mean values of improvements. Maximal inter-incisal opening (MIO), maximal contra-lateral movement (ML) and joint pain using visual analogue scale (VAS) in patients are subjected to arthroscopy or arthrocentesis. Standard deviation values are presented as error bars. (†*P*<0.05; ns, not significant.).

The IAP during lavage was 14.883 ± 0.860 kPa and 13.012 ± 1.021 kPa for the arthroscopic and arthrocentesis groups, respectively. The clinical variables before and after the surgical procedures are shown in Table 1. Both the improvement in MIO (*P*=0.018) and ML (*P*=0.04) after arthroscopic lavage was significantly better than the improvement obtained after arthrocentesis, but pain reduction in the two groups was not statistically different (*P*=0.306). The comparative results of the two groups in terms of MIO and pain improvement are presented in Figure 2. A total of three complications were seen in the entire study group. In the arthroscopic group, one patient presented with a transient frontal palsy (duration 3 months), and one patient developed cervico-facial oedema. In the arthrocentesis group, one cervico-facial oedema was observed. The oedema was probably caused by leakage of the irriagtion fluid from the joint capsule into the deep cervicofascial space. This led to the prolonged intubation (12 h) in order to prevent subsequent post-operative respiratory distress. 

**Table 1:** The mean values of maximal inter-incisal opening (MIO), maximal contra-lateral movement (ML) and joint pain using visual analogue scale (VAS) under arthroscopic lavage and arthrocentesis by Paired-Samples t-test Analysis.

	Arthroscopic Group*				Arthrocentesis Group**			
	MIO (mm)	ML (mm)	VAS (mm)		MIO (mm)	ML (mm)	VAS (mm)	
Before	24.2 ± 4.4	6.1 ± 2.0	60.8 ± 15.9		25.9 ± 5.2	6.9 ± 1.6	53.2 ± 17.5	
After	37.1 ± 4.0	11. 1 ± 1.9	11.9 ± 22.1		35.7 ± 3.8	10.8 ± 2.0	7.3± 9.6	
P values	<0.001	<0.001	<0.001		<0.001	<0.001	<0.001	

* n=37, success rate = 95.49%; **n=41, success rate = 90.24%

**Figure 3:**
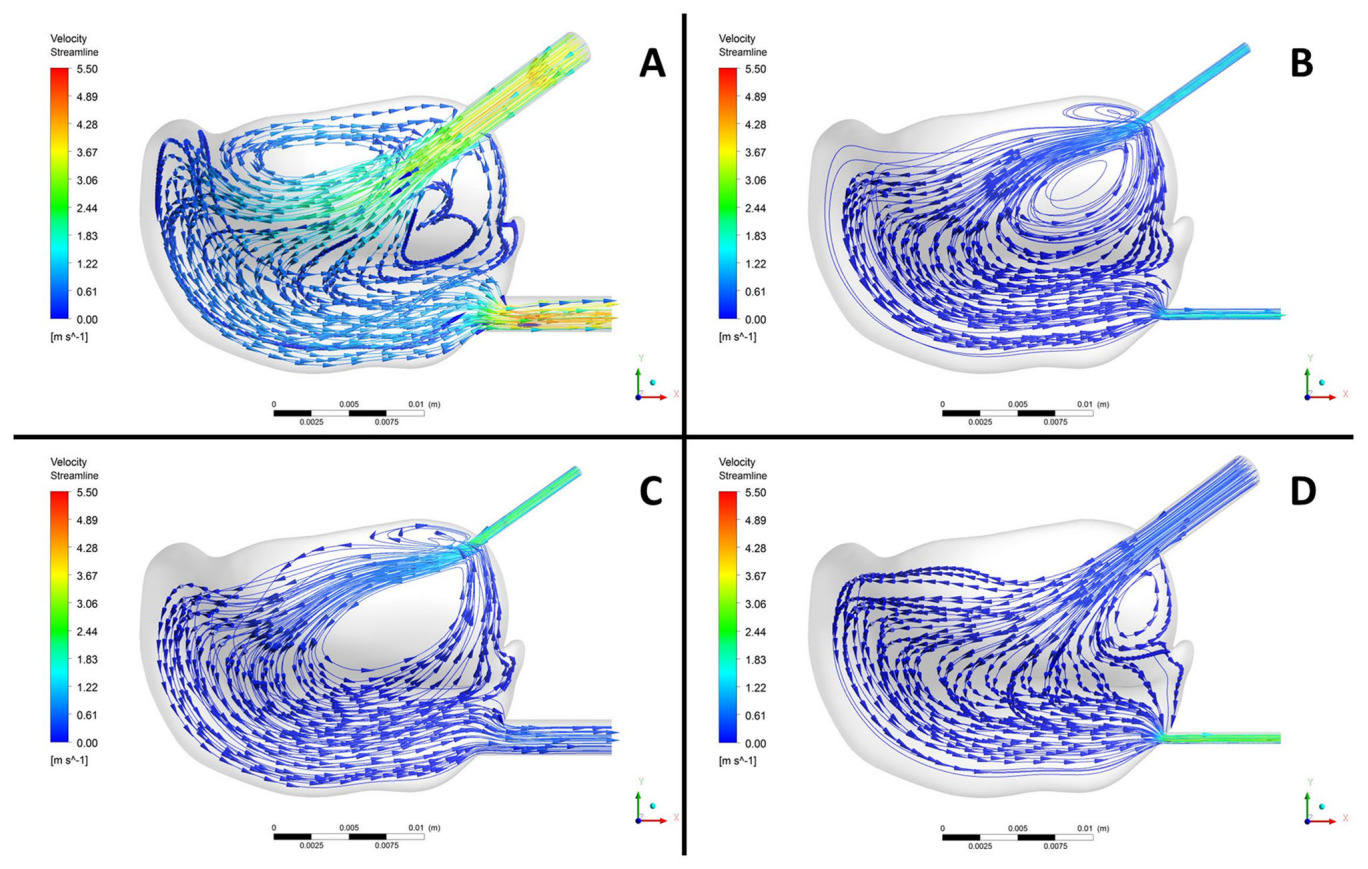
Fluid velocity contours in the upper compartment of a TMJ during a lavage process. The lavage process was conducted with (A) KK, (B) NN, (C) NK and (D) KN type needles. “N” and “K” represent the needle of smaller and bigger diameter, respectively. In acronyms, the former letter represents the inflow needle, and the later one indicates the outflow needle.

**Figure 4:**
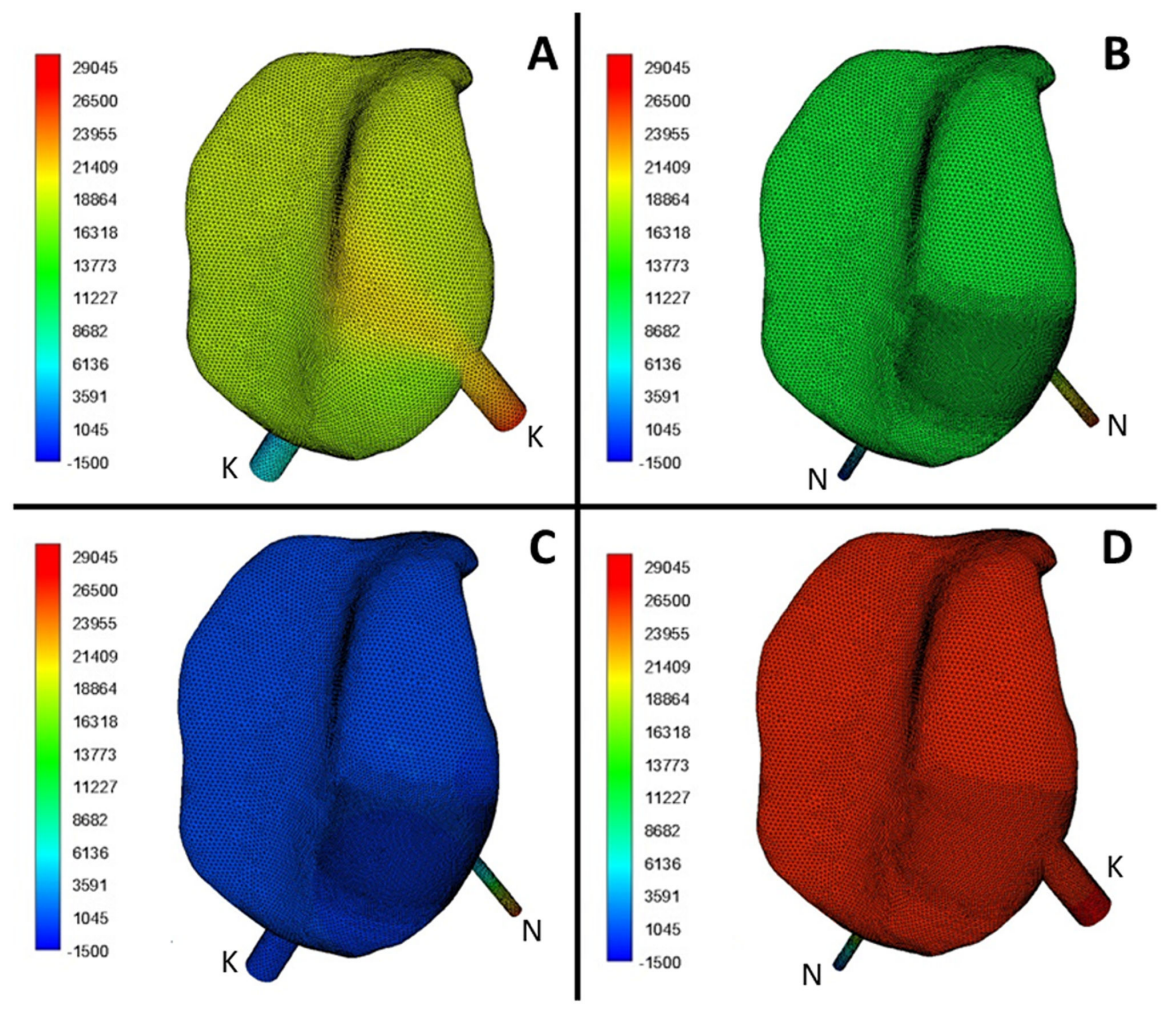
Pressure distribution in the upper compartment of a TMJ during a lavage process. The lavage process was conducted with (A) KK, (B) NN, (C) NK and (D) KN type needles. “N” and “K” represent the needle of smaller and bigger diameter, respectively. In acronyms, the former letter represents the inflow needle, and the later one indicates the outflow needle.

Based on the reconstructed 3D models of the upper compartment of TMJ, the lavage process was simulated and reproduced (Figure 3). The simulations showed that the fluid in the upper compartment displayed a regular flow pattern. The fluid entered the TMJ from the posterolateral needle to the upper compartment, and lashed against the opposite medial wall, the high-pressure area (Figure 4), of the glenoid fossa. The largest part of fluid mass flow near the upper surface of the compartment moved medially along the thicker marginal space of the cavity, reached the anterior space, and then moved to the outflow tube. Only a small part of fluid mass flow reentered the posterior space along the articular wall, joined with the newly injected solution, and subsequently moved out of the joint through outflow cannula. In addition, local vortices were observed at the lower part of the posterior space. 

**Figure 5:**
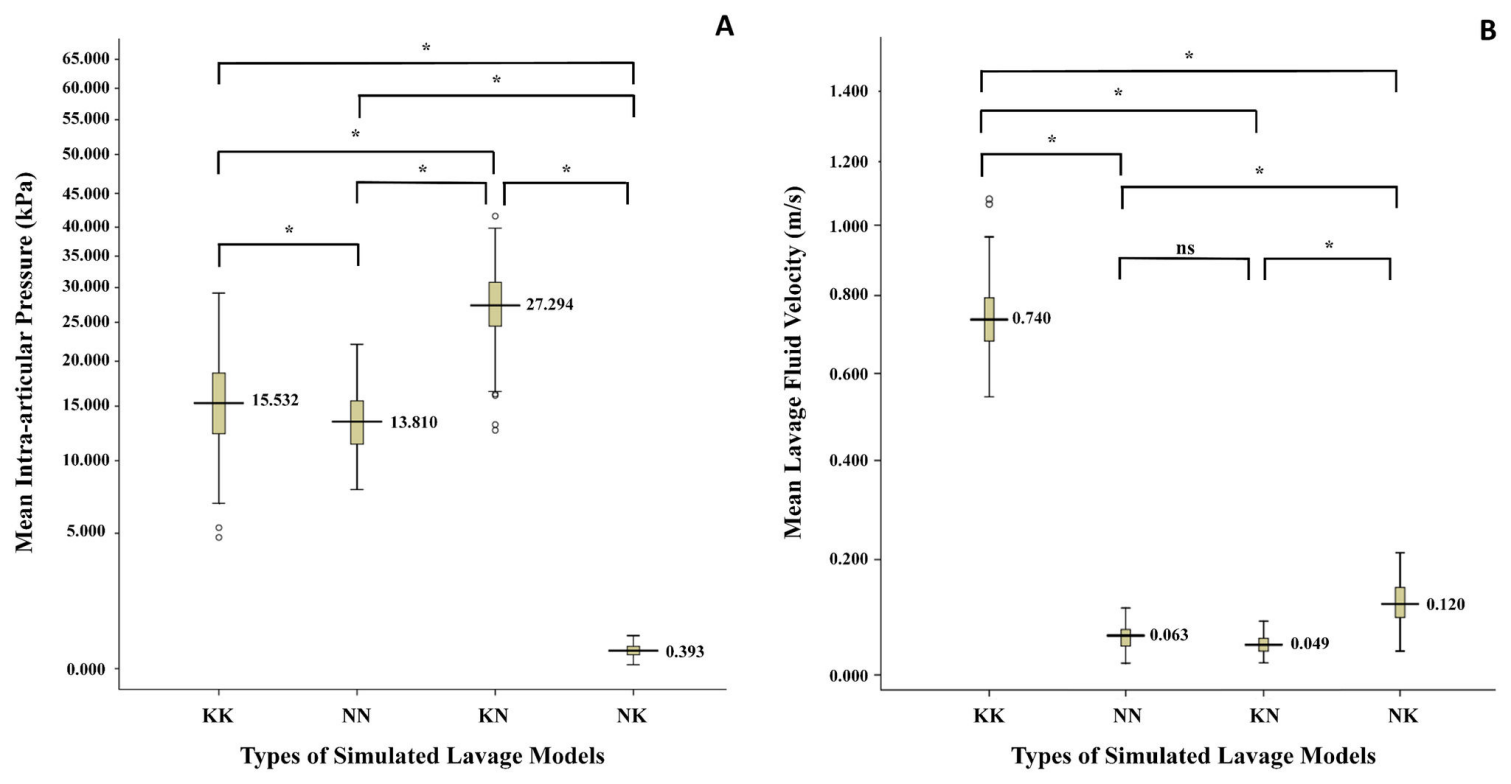
The intra-articular pressure (IAP) and lavage fluid velocity levels in four types of simulated models. (A) The IAP levels were significantly different between four groups (*P*<0.001). (B) The fluid velocity data in KK and NK groups were the two highest types (*P*<0.001), whereas no statistical difference was found between NN and KN groups (*P*=0.306). Standard deviation values are presented as error bars. (*P<0.001; ns, not significant.) .

Analysis of the four numerical models of different lavage processes revealed the insights into IAP and the irrigated flow rate, shown in Figure 5. The IAP levels were highest in KN group (27.294 ± 5.595 m/s; *P*<0.001). In NN group, the fluid velocity data (0.063 ± 0.020 m/s) were lower compared to those in KK group (0.740 ± 0.106 m/s; *P*<0.001) and NK group (0.120 ± 0.038 m/s; *P*<0.001), whereas they did not differ from those in KN group (0.049 ± 0.015 m/s; *P*=0.306).

## Discussion

The role of lavage and accompanying process of arthrolysis has shown excellent success rates in treatment of TMJ disorders. This process has shown to reduce pain and improve joint mobility, sometimes even in patients suffering from advanced stages of degeneration and dysfunction [19-21]. There are two different approaches to lavage and arthrolysis, arthrocentesis and arthroscopic lavage. Various studies have compared the two techniques and have suggested that they vary in terms of prognosis, complications, and long-term outcomes [22-24]. The purpose of this study was to explore the fluid dynamics that exist during these two procedures and find a plausible explanation as to why one technique yields superior results when compared to the other from the perspective of function restoration. The results showed that the different diameter of the needles in TMJ lavage resulted in variable fluid dynamic characteristics, thus contributing to the differential therapeutic effect. 

Retrospective review and analysis of clinical data revealed that the post-operative results in term of MIO, ML and pain reduction statistically improved (*P*<0.001) in both approaches (Table 1). This is consistent with previous research in this arena [25] that has consistently shown that both the arthroscopic lavage and arthrocentesis could provide symptomatic relief and restore jaw function in patients with TMJ-DD. The therapeutic effect, however, greatly depends on whether the irrigated solution could effectively remove the pathological factors, which in turn are closely related to the flow pattern of the lavage fluid. The reconstructed models (Figure 3A & 3B) of the flow pattern associated with these two techniques demonstrated similar flow patterns. The typical pattern was that the solution injected from the posterolateral site lashed against the posterior medial wall of the articular capsule and then the flow split into two channels. The larger portion of the irrigation fluid went out through the anterior space. Only a minor portion moved back to the inflow route, rejoined the rest of fluid, and exited out through the anterior space. The tortuous route of the fluid potentially irrigated all the areas of articular capsule, ensuring a thorough lavage of the cavity [21]. In the narrowest portion, between the anterior slope of the condyle and the posterior slope of the articular eminence, some solution slowed down and formed vortices in the posterior space. The vortices caused the solution flow retardation, with flow rate approaching zero, in the lower part of the posterior space and consequently could not effectively communicate with the major fluid and wash out, resulting in ineffective removal of the pathologic factors, exfoliated cells, and fibrous tissues. Small vortices could also be observed near the outflow site, correlating to the site, angle and depth of the insertion.

When the two groups of arthroscopy and arthrocentesis were compared for clinical outcomes, the arthroscopic lavage was more effective in increasing MIO and ML. Pain reduction in the two groups was not statistically different, though both had improved. A relatively high success rate of 95.49 % was detected in the arthroscopic group, which agreed with what Goudot and Jaquinet [10] had documented in their previous research. Arthroscopic lavage has been advocated as a potentially more effective alternative because the larger diameter portal used in lavage would enable more extensive removal of inflammatory mediators [22]. In our study, the analysis of the current fluid dynamics models demonstrated that the velocity of fluid differed greatly in the two techniques. It was significantly higher in the arthroscopy group at 0.740 ± 0.106 m/s, versus 0.063 ± 0.020 m/s in the arthrocentesis group (*P*<0.001). The lower velocity in the arthrocentesis group may potentially lead to ineffective removal of the catabolites generated from the inflammatory process. A slightly higher IAP value of 15.532 ± 4.951 kPa was obtained in the KK group (arthroscopic group), thus improving the lysis effect of adhesive fibrous tissue [5,26]. These may explain why the therapeutic effect of the arthroscopy is better than that of arthrocentesis, as shown in previous studies as well as our retrospective study.

Although the arthroscopic lavage could yield better therapeutic results, arthrocentesis using smaller lavage needles is considered less traumatic [27] and easier to manipulate [28]. Therefore, for further determination of a suitable apparatus for different clinical uses, situations of different needle diameters as the inflow or outflow routes (i.e. KN, NK, NN and KK group) were simulated in the present study. In this study we observed that the sequence of velocity is: KK>NK>NN~KN, and that there are significant differences between all the groups except the NN and KN groups. Based on these results we can hypothesize that a cannula of a larger diameter in the anterolateral site providing an outflow route could increase the irrigated flow rate. The highest IAP was found in the KN group, then in the KK, NN, and NK group (*P*<0.001 between groups). It can also be concluded that the diameter parameter of both the inflow and outflow needles could affect the IAP during lavage. The therapeutic effect of joint lavage is attributed to the removal of pathologic factors, as well as to the lysis of fibrous tissue with increasing IAP and expansion of the joint cavity. The results suggested that in the severe cavity adhesion cases, always presenting with a limited ability to open the mouth, a larger inflow needle and a smaller outflow needle to increase the IAP may be more effective in ensuring a thorough arthrolysis (Figure 4). The raised IAP ensures that adhesions are lysed and the joint becomes loose, relieving the chief complaints of restriction of movement. In contrast in the cases where the chief complaint is pain, a high flow rate plays a leading role in therapy, so both the large inflow and outflow needles are recommended to elevate the overall flow rate of irrigation fluid.

The IAP was 14.883 ± 0.860 kPa during the arthroscopic lavage and 13.012 ± 1.021 kPa during arthrocentesis, slightly less than the theoretical value in the simulated situations, which may due to the elasticity of the cavity capsule instead of the rigid reconstruction employed for simulation process. The capsule wall was set to be rigid in order to easily derive changes in IAP. Therefore, the calculated pressures present may not completely represent those occurring *in vivo* in TMJ-DD cases. When the lavage pressure increases beyond the limits of compensative ability of the capsule and the elasticity of the capsule wall, it leads to severe deformation of the capsule, and this numerical model with rigid wall hypothesis would not be suitable for analyzing the fluid dynamics.

In the present study, we found that the diameter of lavage needles could affect the irrigated flow rate and IAP during lavage. Thus, we suggest that to improve the clinical outcomes of TMJ-DD, surgical apparatus should be chosen according to the symptoms and clinical examination, including the primary symptom and also the radiography findings of the patients. This study likely represents the first research to elucidate the difference of clinical outcomes with biomechanical investigation into IAP and irrigated flow rate during the joint lavage operation. We hope that our findings will improve the understanding of the fluid dynamic mechanisms of TMJ lavage, and ultimately will provide strategies for the alternative treatment for TMJ-DD. 

## References

[1] Kropmans TJ, Dijkstra PU, Stegenga B, de Bont LG (1999) Therapeutic outcome assessment in permanent temporomandibular joint disc displacement. J Oral Rehabil 26(5): 357-363. doi:10.1046/j.1365-2842.1999.00417.x. PubMed: 10373081. 10373081

[2] Ohnishi M (1990) Arthroscopy and arthroscopic surgery of the temporomandibular joint (T.M.J.). Rev Stomatol Chir Maxillofac 91(2): 143-150. PubMed: 2309088. 2309088

[3] Nitzan DW (2006) Arthrocentesis--incentives for using this minimally invasive approach for temporomandibular disorders. Oral Maxillofac Surg Clin North Am 18(3): 311-328. doi:10.1016/j.coms.2006.03.005. PubMed: 18088835. 18088835

[4] Monje-Gil F, Nitzan D, González-Garcia R (2012) Temporomandibular joint arthrocentesis. Review of the literature. Med Oral Patol Oral Cir Bucal 17(4): e575-e581. PubMed: 22322493. 22322493 10.4317/medoral.17670 PMC3476018

[5] van Oosterhout M, Sont JK, Bajema IM, Breedveld FC, van Laar JM (2006) Comparison of efficacy of arthroscopic lavage plus administration of corticosteroids, arthroscopic lavage plus administration of placebo, and joint aspiration plus administration of corticosteroids in arthritis of the knee: A randomized controlled trial. Arthritis Rheum 55(6): 964-970. doi:10.1002/art.22340. PubMed: 17139644. 17139644

[6] Barkin S, Weinberg S (2000) Internal derangements of the temporomandibular joint: the role of arthroscopic surgery and arthrocentesis. J Can Dent Assoc 66(4): 199-203. PubMed: 10789172. 10789172

[7] Rehman KU, Hall T (2009) Single needle arthrocentesis. Br J Oral Maxillofac Surg 47(5): 403-404. doi:10.1016/j.bjoms.2008.09.014. PubMed: 18977563. 18977563

[8] Guarda-Nardini L, Manfredini D, Ferronato G (2010) Short-term effects of arthrocentesis plus viscosupplementation in the management of signs and symptoms of painful TMJ disc displacement with reduction. A pilot study. Oral Maxillofac Surg 14(1): 29-34. doi:10.1007/s10006-009-0179-z. PubMed: 19821126. 19821126

[9] Machon V, Hirjak D, Lukas J (2011) Therapy of the osteoarthritis of the temporomandibular joint. J Cranio-Maxillofac Surg 39(2): 127-130. doi:10.1016/j.jcms.2010.04.010. PubMed: 20692843. 20692843

[10] Goudot P, Jaquinet AR, Hugonnet S, Haefliger W, Richter M (2000) Improvement of pain and function after arthroscopy and arthrocentesis of the temporomandibular joint: a comparative study. J Cranio-Maxillofac Surg 28(1): 39-43. doi:10.1054/jcms.1999.0103. PubMed: 10851672. 10851672

[11] Dolwick MF, Dimitroulis G (1994) Is there a role for temporomandibular joint surgery? Br J Oral Maxillofac Surg 32(5): 307-313. doi:10.1016/0266-4356(94)90052-3. PubMed: 7999739. 7999739

[12] Dworkin SF, LeResche L (1992) Research diagnostic criteria for temporomandibular disorders: review, criteria, examinations and specifications, critique. J Craniomandib Disord 6(4): 301-355. PubMed: 1298767. 1298767

[13] Sanromán JF (2004) Closed lock (MRI fixed disc): a comparison of arthrocentesis and arthroscopy. Int J Oral Maxillofac Surg 33(4): 344-348. doi:10.1016/j.ijom.2003.10.005. PubMed: 15145035. 15145035

[14] Alkan A, Baş B (2007) The use of double-needle canula method for temporomandibular joint arthrocentesis: clinical report. Eur J Dent 1(3): 179-182 PubMed : 19212563 PMC2638237 19212563

[15] Kaneyama K, Segami N, Nishimura M, Sato J, Fujimura K et al. (2004) The ideal lavage volume for removing bradykinin, interleukin-6, and protein from the temporomandibular joint by arthrocentesis. J Oral Maxillofac Surg 62(4): 657-661. PubMed: 15170274. 15170274 10.1016/j.joms.2003.08.031

[16] Xu Y, Zhan J, Zheng Y, Han Y, Zhang Z et al. (2012) Synovial fluid dynamics with small disc perforation in temporomandibular joint. J Oral Rehabil 39(10): 719-726. doi:10.1111/j.1365-2842.2012.02307.x. PubMed: 22582815. 22582815

[17] Xu Y, Zhan JM, Zheng YH, Han Y, Zhang ZG et al. (2012) Computational synovia dynamics of a normal temporomandibular joint during jaw opening. J Formosan Medical 112(6): 346-351. 10.1016/j.jfma.2012.02.015 23787012

[18] Batchelor GK (2000) An Introduction to Fluid Dynamics. London: Cambridge University Press.

[19] González-García R, Rodríguez-Campo FJ (2011) Arthroscopic lysis and lavage versus operative arthroscopy in the outcome of temporomandibular joint internal derangement: a comparative study based on Wilkes stages. J Oral Maxillofac Surg 69(10): 2513-2524. doi:10.1016/j.joms.2011.05.027. PubMed: 21939814. 21939814

[20] Zhu Y, Zheng C, Deng Y, Wang Y (2012) Arthroscopic surgery for treatment of anterior displacement of the disc without reduction of the temporomandibular joint. Br J Oral Maxillofac Surg 50(2): 144-148. doi:10.1016/j.bjoms.2011.02.004. PubMed: 21377774. 21377774

[21] Leibur E, Jagur O, Müürsepp P, Veede L, Voog-Oras U (2010) Long-term evaluation of arthroscopic surgery with lysis and lavage of temporomandibular joint disorders. J Cranio-Maxillofac Surg 38(8): 615-620. doi:10.1016/j.jcms.2010.02.003. PubMed: 20335040. 20335040

[22] Hobeich JB, Salameh ZA, Ismail E, Sadig WM, Hokayem NE et al. (2007) Arthroscopy versus arthrocentesis. A retrospective study of disc displacement management without reduction. Saudi Med J 28(10): 1541-1544. PubMed: 17914517. 17914517

[23] Fridrich KL, Wise JM, Zeitler DL (1996) Prospective comparison of arthroscopy and arthrocentesis for temporomandibular joint disorders. J Oral Maxillofac Surg 54(7): 816-820; discussion: 10.1016/S0278-2391(96)90526-1. PubMed: 8676225. 8676225

[24] Murakami K, Hosaka H, Moriya Y, Segami N, Iizuka T (1995) Short-term treatment outcome study for the management of temporomandibular joint closed lock. A comparison of arthrocentesis to nonsurgical therapy and arthroscopic lysis and lavage. Oral Surg Oral Med Oral Pathol Oral Radiol Endod 80(3): 253-257. doi:10.1016/S1079-2104(05)80379-8. PubMed: 7489265. 7489265

[25] Sakamoto I, Yoda T, Tsukahara H, Imai H, Enomoto S (2000) Comparison of the effectiveness of arthrocentesis in acute and chronic closed lock: analysis of clinical and arthroscopic findings. Cranio 18(4): 264-271. PubMed: 11202846. 11202846 10.1080/08869634.2000.11746140

[26] Morey-Mas MA, Caubet-Biayna J, Varela-Sende L, Iriarte-Ortabe JI (2010) Sodium hyaluronate improves outcomes after arthroscopic lysis and lavage in patients with Wilkes stage III and IV disease. J Oral Maxillofac Surg 68(5): 1069-1074. doi:10.1016/j.joms.2009.09.039. PubMed: 20144496. 20144496

[27] Hosaka H, Murakami K, Goto K, Iizuka T (1996) Outcome of arthrocentesis for temporomandibular joint with closed lock at 3 years follow-up. Oral Surg Oral Med Oral Pathol Oral Radiol Endod 82(5): 501-504. doi:10.1016/S1079-2104(96)80193-4. PubMed: 8936512. 8936512

[28] Adachi PL, Kaba SP, Martins MT, Hueb CH, Shinohara EH (2008) Arthrocentesis in the treatment of loose bodies of the temporomandibular joint associated with synovial chondromatosis. Br J Oral Maxillofac Surg 46(4): 320-321. doi:10.1016/j.bjoms.2007.07.208. PubMed: 17920736. 17920736

