# Correction: A Comparative Study between Use of Arthroscopic Lavage and Arthrocentesis of Temporomandibular Joint Based on Computational Fluid Dynamics Analysis

**DOI:** 10.1371/annotation/3b3b7d2a-9839-458e-906a-4969971f156a

**Published:** 2014-01-06

**Authors:** Yue Xu, Han Lin, Ping Zhu, Wenyan Zhou, Yi Han, Youhua Zheng, Zhiguang Zhang

Affiliation number 1 for the first, second, third, sixth and seventh authors is incorrect. The correct affiliation is: Guanghua School of Stomatology, Guangdong Provincial Key Laboratory of Stomatology, Sun Yat-sen University, Guangzhou, Guangdong, People’s Republic of China.

